# AAPM Medical Physics Practice Guideline 1.b: CT protocol management and review practice guideline

**DOI:** 10.1002/acm2.13193

**Published:** 2021-05-03

**Authors:** Dianna D. Cody, Chad M. Dillon, Tyler S. Fisher, Xinming Liu, Michael F. McNitt‐Gray, Vikas Patel

**Affiliations:** ^1^ U.T.M.D Anderson Cancer Center Houston TX USA

**Keywords:** CT oversight, CT parameters, CT protocols

## Abstract

The American Association of Physicists in Medicine (AAPM) is a nonprofit professional society whose primary purposes are to advance the science, education and professional practice of medical physics. The AAPM has more than 8000 members and is the principal organization of medical physicists in the United States. The AAPM will periodically define new practice guidelines for medical physics practice to help advance the science of medical physics and to improve the quality of service to patients throughout the United States. Existing medical physics practice guidelines will be reviewed for the purpose of revision or renewal, as appropriate, on their fifth anniversary or sooner. Each medical physics practice guideline represents a policy statement by the AAPM, has undergone a thorough consensus process in which it has been subjected to extensive review, and requires the approval of the Professional Council. The medical physics practice guidelines recognize that the safe and effective use of diagnostic and therapeutic radiology requires specific training, skills, and techniques, as described in each document. Reproduction or modification of the published practice guidelines and technical standards by those entities not providing these services is not authorized. The following terms are used in the AAPM practice guidelines: (a) Must and Must Not: Used to indicate that adherence to the recommendation is considered necessary to conform to this practice guideline. (b) Should and Should Not: Used to indicate a prudent practice to which exceptions may occasionally be made in appropriate circumstances.

## 1 INTRODUCTION

The review and management of computed tomography (CT) protocols are a facility’s ongoing mechanism of ensuring that exams being performed achieve the desired diagnostic image quality while optimizing radiation dose and properly exploiting the capabilities of the equipment being used. Therefore, protocol management and review are essential activities in ensuring patient safety and acceptable image quality. While several states require accreditation that calls upon these activities, the activities themselves have been explicitly identified as essential by one state,^1^ regulatory and accreditation groups such as the American College of Radiology (ACR) CT Accreditation program and Intersocietal Accreditation Commission (IAC),^2^, ^3^, ^4^ as well as the Joint Commission in its requirements for diagnostic imaging services among others.^5^ The AAPM considers these activities to be essential to any quality assurance (QA) program for CT, and as an ongoing investment in improved quality of patient care.

CT exam protocols are used to obtain the diagnostic image quality required for the exam, while optimizing radiation dose to the patient and ensuring the proper utilization of the scanner features and capabilities. Protocol review refers to the periodic evaluation of all aspects of CT exam protocols. These parameters include acquisition parameters (e.g., kVp), patient instructions (e.g., breathing instructions), the administration and amounts of contrast material (intravenous, oral, etc.), and postprocessing parameters. Protocol management refers to the process of review, implementation, and verification of protocols within a facility’s practice.

This is a complex undertaking in the present environment. The challenges in optimization of dose and image quality are compounded by the lack of a commercially available, vendor‐neutral automated mechanism to collect and modify protocols systemwide.^6^ The manual labor involved in identifying, recording, and compiling for review and subsequent implementation all relevant parameters of active protocols is not inconsequential.^7^, ^8^ The clinical community needs effective protocol management tools and efficient methods to replicate protocols across different scanners in order to ensure consistency.^9^, ^10^ The ability to quickly view and understand the myriad of CT protocol parameters contained within a single exam type is critical to the success of protocol review. The ability to quickly identify an outlier protocol parameter would also be extremely beneficial to the CT protocol review process.

This MPPG applies only to CT scanners used for diagnostic imaging. However, some elements of protocol review described in this document are also appropriate for:

- CT scanners used only for therapeutic radiation treatment planning;
- CT scanners used only for calculating attenuation correction coefficients for nuclear medicine studies;
- 3D angiography and flat panel CT systems;
- Dental CT units; and
- Interventional CT scanners

## 2 DEFINITIONS

- CT protocol — The collection of values of the user configurable parameters, and technologist instructions to perform an imaging study.^11^, ^12^ Protocols may be relatively simple for some body part specific systems or highly complex for full‐featured, general purpose CT systems.^13^
- Qualified medical physicist — as defined by AAPM Professional Policy 1^14^
- CT protocol management and review system — The personnel (“team”), documents, procedures, and software used to manage and review CT protocols.

## 3 STAFFING QUALIFICATIONS AND RESPONSIBILITIES

### 3.1 The protocol review and management team

Protocol review and management requires a team effort; this team **must** consist of at least a lead CT radiologist, the lead CT technologist, and qualified medical physicist (QMP). A senior member of the facility administration team **should** also be involved. This could be the chief medical or administrative officer for the facility, or a dedicated radiology department administrator/manager, as determined by hospital leadership. If a senior member of the facility administration team is not a member of the protocol review and management team, there **should** be a clear delineation of the reporting structure.

This team **must** be responsible for protocol design and review of important parameter settings. Each team member brings different expertise and may have different responsibilities in the protocol review and management process. To be successful, it is very important that the expectations of roles and responsibilities of each member are clearly described. Each member of the group must be able to work together as a team. The flow chart in Appendix A is an example of how team members **should** work together and in parallel during the process.^7^ Additional examples of protocol management based on individual facilities’ experience are discussed in Refs. [8], [15], and [16]. The team members, their qualifications, and expectations are described below.

#### 3.1.1 Qualified medical physicist (QMP)

The first professional policy of the AAPM provides a comprehensive definition of a qualified medical physicist (QMP).^14^ The subfield of medical physics applicable for CT protocol management is Diagnostic Medical Physics. As stated by the Policy, “a [QMP] is an individual who is competent to independently provide clinical professional services in one or more of the subfields of medical physics.”

#### 3.1.2 Responsibilities of the QMP

In the context of CT protocol management and review, the QMP’s responsibilities may vary, depending on the type of facility being supported; regardless, the QMP **must** be involved in the review of all protocols. These considerations **should** be balanced with adequate response times to facility inquiries.

A QMP’s time at a facility **should** include but not be limited to:

- meeting with the CT protocol management and review team;
- clinical observation;
- phantom measurements if appropriate;
- side‐by‐side image review with radiologist(s);
- artifact review with technologist(s) and/or radiologist(s); and
- discussion of equipment performance and operation, etc.

While regular dialogue is important, the lead CT radiologist **should** lead the CT protocol management and review process; the QMP is an integral member of the team. The QMP may elect to perform baseline dose measurements and image quality tests for any system where this information is not already available to the QMP.

#### 3.1.3 In‐house QMP

For the in‐house QMP, this ongoing CT protocol review project may consume much of his/her time, so the QMP **should** be sure to adequately communicate with his/her supervisor(s), with other team members, and with department/hospital management in this regard.^17^ The facility **should** understand that the CT protocol management and review process is an ongoing investment in improved quality and safety of patient care.

In‐house QMPs may be able to arrange more frequent meetings with CT protocol management and review team members than their consulting colleagues; six to 12 meetings annually may be more appropriate for facilities with in‐house QMPs. Meeting frequency can be expected to increase when new CT scanner models and/or major CT technology improvements are implemented.

#### 3.1.4 Consulting QMP

It is important to note that CT protocol management and review services are above and beyond normal QMPs consulting services (e.g., the annual physics survey), which have traditionally been limited to image quality, dosimetry, and basic protocol review for a few selected examinations. Consultant QMPs **should** make this clear to their clients and negotiate their services appropriately.

QMPs providing consulting services **should** maintain regular dialogue with the facility via convenient means (e.g., email and phone). It may be beneficial to use a communication process that provides a log of these interactions. It is recommended that the consulting QMP discusses with each facility access to images, including, but not limited to, remote access to the facility’s picture archiving and communication system (PACS) for improved consultative capabilities.

Consulting QMPs **should** work with the facility to arrange mutually agreeable times to visit the facility for CT protocol review activities. Three to four visits annually may be reasonable. Protocol review activities may be performed remotely depending on the agreement of the facility and the availability of remote access to PACS and/or automated dose reporting software. However, it is recommended that QMP **should** visit the facility three to four times in the first year of the review process being implemented to perform CT protocol review activities.

#### 3.1.5 Qualifications and expectation of the lead CT technologist

The American Society of Radiologic Technologists (ASRT) has developed a practice standard entitled *The Practice Standards for Medical Imaging and Radiation Therapy — Computed Tomography Practice Standards,* effective June 19, 2011, which describes the education and certification requirements and scopes of practice for CT technologists.^18^

The lead CT technologist is expected to provide the interface between the patient, staff, and the equipment. This includes workflow, the assembly and management of the CT protocols, and education of the technologists.

#### 3.1.6 Qualifications of the CT radiologist

Facilities should refer to the ACR for guidance on physician qualification requirements. These are outlined in *Practice Guideline for Performing and Interpreting CT* ^19^ and *CT Accreditation Program Requirements*.^20^

The CT radiologist leads the CT protocol management and review and defines image quality requirements.^21^ For facilities with a large number of radiologists, where no single radiologist is willing to take responsibility for reviewing all CT protocols, the facility is expected to arrange radiologist review as appropriate. This could take the form of a rotating assignment or something similar.

#### 3.1.7 Responsibilities of the administrative team member

An administrative team member’s time at a facility **should** include but not be limited to:

- allocating resources;
- providing administrative support for the protocol review and management team, including releasing people from their clinical scheduled duties in order to attend meetings;
- providing administrative support for meetings, including scheduling, remote phone access, and documentation of proceedings
- providing utilization data and quantitative metrics as requested by the protocol review and management team; and
- analyzing project value and promoting results.

A more comprehensive, process‐oriented chart of these responsibilities can be found in Appendix A.

## 4 THE PROTOCOL MANAGEMENT AND REVIEW PROCESS

It is important that the CT protocol review and management team designs and reviews all new or modified protocol settings for existing and new scanners to ensure that both image quality and radiation dose aspects are appropriate. Each member of CT protocol review and management team has a critical role related to his or her specific area of expertise for the evaluation, review, and implementation of protocols. The following elements **should** be considered for inclusion in a specific facilities’ protocol review process:

- During the review process, the CT protocol management team **should** be aware of new and innovative technologies that can further improve image quality or better optimize patient dose.
- Particular attention **should** be paid to the specific capabilities of each individual scanner (e.g., minimum rotation time, automatic exposure controls including both tube current modulation, as well as kV selection technologies, iterative reconstruction, reconstruction algorithms, etc.) to ensure maximum performance of the system is achieved. In addition, consideration **should** be made to consolidate protocols or remove legacy protocols that are no longer current or applicable.
- The review process **should** include a review of the most current literature such as ACR practice guidelines,^19^ AAPM protocol list,^13^ and peer‐reviewed journals, etc., to ensure state‐of‐the‐art protocols are being utilized.

The following considerations are important during protocol review:

- **Recommendations for State and National Guidance**
  Laws and regulations can vary greatly by region and state. The QMP **must** be familiar with applicable federal law and the specific requirements for the state or local jurisdiction where the facility is located. Protocol review and management, while not always explicitly required, may often facilitate compliance with provisions relating to radiation dose in CT. Links to applicable state regulations can be found at: http://www.aapm.org/government_affairs/licensure/default.asp.
- **Frequency of Review**
  The review process **must** be consistent with federal, state, and local laws and regulations. If there is no specific regulatory requirement, the frequency of all protocols reviewed **should** be no less frequent than 24 months. This review **should** include all new protocols added since the last review. However, the best practice would be to review a facility’s most frequently used protocols at least annually. Protocols should be reviewed as they are being developed on new platforms.
- **Clinically Significant Protocols that Require Annual Review**
  Clinically significant protocols include those that are used frequently or have the potential to result in significant patient dose. These include the following six protocols. Facilities that do not perform all of the exams listed below **must** select additional protocols at their facility, either the most frequently performed, higher dose, or screening protocols, to a total of at least six for annual review. The six clinical protocols requiring annual review are:
  - Pediatric head (1 yr old) (if performed at the institution)
  - Pediatric abdomen (5 yr old; 40–50 lb. or approx. 20 kg) (if performed at the institution)
  - Adult head
  - Adult abdomen (70 kg)
  - High resolution chest
  - Brain perfusion (if performed at the institution)
- **Protocol Naming**
  The primary focus should be on consistent names across the enterprise.^22^ A facility should consider naming CT protocols in a manner consistent with the RadLex Playbook ID, if participating in the Dose Index Registry.^23^, ^24^ This would provide a more consistent experience for patients and referring physicians, and allow more direct comparison among various facilities. This practice may also allow more direct utilization of the ACR Dose Index Registry^24^ tools and provide more efficient automated processes with postprocessing workstations. Also, the standardization of protocol names between scanners, even when the scanners are of different makes and models, is strongly encouraged. Appropriate protocol naming will likely result in fewer technologist errors and allow more efficient comparison of protocol parameters between scanners.
- **Permissions**
  - It is important that each facility establishes a process for determining who has permission to access the protocol management systems. Each facility **should** decide and document who has permission to change protocol parameters on the scanner(s). If the scanner allows password protection of protocols, then the facility is encouraged to use this important safety feature. Facilities **should** also decide how passwords are protected and archived.
  - Each facility should decide on the process of making protocol adjustments and the frequency with which these adjustments should be made. This includes decisions as to what approvals need to be secured before a protocol adjustment may be made, and the documentation process (e.g., a change control log documenting the rationale for each change, as well as who authorized or motivated the change).
  - Each facility should consider how to most effectively utilize the NEMA XR 26 standard (Access Controls for Computed Tomography)^25^ when these tools become available on scanners at their facility.
- **Acquisition parameters** including kV, mA, rotation time, collimation or detector configuration, pitch, etc., should be reviewed to ensure they are appropriate for the diagnostic image quality (noise level, spatial resolution, etc.) necessary for the clinical indication(s) for the protocol, while optimizing radiation dose. For example, a slow rotation time and/or low pitch value would not be appropriate for a chest CT exam due to breath‐hold issues. The acquisition parameters for special exams such as dual or multi‐energy, or high spatial resolution mode, may require special attention.
- **Reconstruction parameters** such as the width of the reconstructed image (image thickness), distance between two consecutive reconstructed images (reconstruction interval), reconstruction algorithm/kernel/filter, and the use of additional image planes (e.g., sagittal or coronal planes, etc.) **should** also be reviewed to ensure appropriate diagnostic image quality (noise level, spatial resolution, etc.) necessary for the clinical indication(s) for the protocol. For example, a high‐resolution chest exam typically generates thin (~1 mm) images using a sharp reconstruction filter. Series description labels may depend on PACS hanging protocol codes.
- **Advanced dose reduction techniques should be considered** when the use of such techniques is consistent with the goals of the exam. Depending on the capabilities of each specific scanner, consider use of the following, if they are available:
  - Automatic exposure control (e.g., tube current modulation, automatic kV selection, or organ dose modulation) methods.
  - Iterative reconstruction techniques.
  - **Acquisition parameters should be adjusted for patient size**, either through a series of manual adjustments or through the use of automatic techniques (such as tube current modulation methods that adjust for patient size).
- **Radiation dose management tools** fall under two related but different categories, and may provide CT dose data that can be used to determine facility reference dose ranges.
  - Radiation dose management tools that identify when potentially high‐radiation dose scans are being prescribed **should** be implemented when available. The first type of radiation dose management tools may exist on the scanner and can alert the user, prior to the scan, that the prescribed protocol exceeds the established Notification Value. Examples and methods are described in the NEMA XR25 standard (e.g., Dose Check).^26^ These should be implemented and used when available.^27^ It is recommended that the associated Notification Value is established and/or confirmed during the review process. The Alert Value should be established during the scanner acceptance process by the protocol review and management team.^26^
  - A second type of radiation dose management tool may be used to retrospectively monitor dose metrics from clinical exams.^28^ Statistical analysis of dose parameter values for a specific exam or clinical indication (e.g., average CTDIvol for a routine noncontrast head) can be provided. Participation in a national registry (such as the ACR Dose Index Registry)^24^ and use of commercial dose tracking products are now available for this purpose. Size‐specific dose estimates (SSDE) and/or water‐equivalent diameter (WED) can be considered for comparison of dose metrics if available during protocol review. Utilization of radiation dose structured report (RDSR) and the patient RDSR if available is included.
**Note**: These dose management systems may use dose alert terminology that is distinct from the Dose Check^26^ terminology described above in Section 4.i.i.
  - The facility should review CTDIvol values from clinical exams. When external benchmarks are available, a facility's CTDIvol values should be compared to reference values of the ACR CT Accreditation Program,^2^ published Diagnostic Reference Levels,^29^, ^30^ or AAPM CT protocols.^13^
**Note**: These reference values may be exceeded for individual patient scans (such as for a very large patient, or when the routine protocol is not used because of a different clinical indication, or when the reference value only refers to a single pass in a multipass study).
- **Populating Protocols Across Scanners**
  Each facility **should** decide on the process by which protocol parameters are populated across additional scanners (whether this is done manually or by copy/paste, if the scanners allow). The facility **should** decide whether there are “master” or “primary” scanners in the facility where manual protocol adjustments are to be made and archived, and that set of protocols moved to the other similar scanners, or if another strategy will be employed.
- **Documentation**
  The CT protocol review and management team **should** maintain documentation of all changes to protocols, and historical protocols **should** be available for review. Documentation should include the rationale for changes (e.g., improve temporal resolution, reduce breath‐hold time, reduce patient dose, etc.). The latest protocol **should** be readily and obviously available to users during clinical protocol selection. In some settings, it may be helpful to maintain historical protocols on the scanner, in a less conspicuous location or clearly labeled as a legacy protocol.
  The facility should decide and document who is responsible for maintaining the overall protocol description documentation. The facility **should** also describe whether the protocol description documentation is accessible to others for reference, how often it is updated, and how all protocols (on the scanners as well as the protocol description documentation) are archived.
- **Periodic Vendor‐specific Education/Refresher Sessions**
  The CT protocol review and management team is responsible for ensuring that each member is adequately trained for protocol review on each scanner used at the facility. Each member of the CT protocol review and management team **should** receive refresher training no less than annually or when new technology is introduced that substantially impacts image quality or dose to the patient.
  - Available educational resources **should** be considered in order to keep staff updated on current best practices.
  - Periodic refresher training **should** be scheduled for all members of the CT protocol review and management team.
  - Attendance **should** be taken at initial and all refresher training sessions, and consequences identified for failure to complete training.
- **Verification**
  Once a CT protocol review and management process has been established, the CT protocol review and management team **must** institute a regular review process such that all summary protocol documents match the installed version on the scanners, and to be sure that no unintended changes have been applied that may degrade image quality or unreasonably increase dose.
  As a best practice, the CT protocol review and management team **should** conduct a random survey of performed specific exam types to verify that the protocols used are acceptable and consistent with protocols specified above. This **should** involve a limited review of recent patient cases to assess:
  - Acquisition and reconstruction parameters,
  - Image quality, and
  - Radiation dose.

## 5 CONCLUSION

Computed tomography protocol management and review is an important part of a CT facility’s operation and is considered required by many state regulatory bodies, accrediting, and professional organizations. Protocol parameter control and periodic review will help maintain the facility’s image quality to acceptable levels, and will serve to assure patient safety and continuous improvement in the imaging practice.

## 6 Disclosure Statement

The Chair of Task Group No. 326 ‐ MPPG 1.b: CT Protocol Management and Review (TG326) has reviewed the required Conflict of Interest statement on file for each member of TG326 and determined that disclosure of potential Conflicts of Interest is an adequate management plan. Disclosures of potential Conflicts of Interest for each member of TG326 are found at the close of this document.

## APPENDIX A

Example of how team members may work together and in parallel during the process.^6^
